# Abstract of a Paper on Duodenal Ulcer and Its Treatment

**Published:** 1904-12-03

**Authors:** D'Arcy Power

**Affiliations:** Surgeon to St. Bartholomew's Hospital


### ABSTRACT OF A PAPER ON DUODENAL ULCER AND ITS TREATMENT.

Read at the Medical Society of London, November 28th, 1904, by D'Arcy Power, F.R.C.S.Eng., Surgeon to St. Bartholomew's Hospital.

Mr. D'Arcy Power said that he thought the subject of duodenal ulcer had not yet received adequate attention but that as during the last few years he had operated upon seven cases and had had the opportunity of seeing several others, he felt justified in bringing the condition under the notice of the Fellows of the Medical Society of London.

He divided the cases of duodenal ulcer into two great groups, those which perforated and those which did not perforate, and gave the following pictures of the two classes.

The subject of a perforating duodenal ulcer is usually a man in the prime of life who has hitherto considered himself in perfect health, or who has suffered, if at all, from only the slightest symptoms of indigestion. Without warning he is suddenly seized with a stomach-ache of such severity that he becomes collapsed and sends at once for assistance. He may vomit; but from the onset of the pain he passes neither flatus nor fæces. Examination within an hour or two of the attack shows him lying on his back, afraid to move, his breathing shallow and very rapid; his pulse small, regular and quick but not nearly so much accelerated as his respiration. Temperature normal. He looks pinched and anxious, and is unable to localise his pain but complains that it is worse along the upper half and down the right side of the abdomen. The stomach is not distended and is not motionless though it moves less freely than it should do during respiration. It is held somewhat rigid, the muscles on the right side being rather more tonically contracted than those on the left. It is everywhere tender and tympanitic. The area of liver dulness may or may not be altered, and there is sometimes a point of maximum tenderness in the right hypochondrium.

If no operation be performed the pain gets less acute, but even more generalised than it was at first. Later still it is localised in the right iliac region which may become especially tender, full, and motionless, whilst the rest of the abdomen moves during respiration, so that the case may easily be mistaken for one of appendicitis. More usually, however, the abdomen becomes uniformly distended and the patient who has rallied from the initial shock is again collapsed and dies of acute peritonitis.

Mr. Power then continued: "Now look at a case where there has been a duodenal ulcer which did not perforate. It is even more interesting than the last, for the diagnosis is more difficult, and the sequelæ are more remote.

"The patient is an older man, perhaps between 50 and 60. Thin and haggard, he tells you that he is a martyr to indigestion, and that for months past he has suffered atrocious pain in his stomach, which is relieved by vomiting He has dieted himself in every possible manner, he has made all kinds of local applications to his abdomen, he has visited all sorts of watering-places, and he has gone in vain from one physician to another seeking a cure. Examination shows him to be a mere bag of bones, constipated, with cold extremities and a listless dejected aspect. His abdomen is loose, the subcutaneous veins may be enlarged, and there is visible peristalsis from left to right in the epigastric region. Percussion tells you that the stomach is greatly dilated, and it is not very unusual to feel a lump in the neighbourhood of the pylorus. For a moment you think of cancer of the pylorus or gall-bladder and you question the patient a little more closely. He is sure that he has been suffering for years, for so long in fact that he hardly recollects the beginning of his trouble. A few well directed inquiries may elicit that many years ago when he was a young man he once or twice brought up a large quantity of blood without serious pain or discomfort, or that he had an illness which no one seemed to know much about. He was treated for gall-stones, or appendicitis, or simply for 'liver.' The attack was painful and kept him in bed, but the exact details have passed from his mind and for some years he was as healthy a man as ever.

"This is a case of duodenal obstruction due to cicatrisation of an old ulcer, the irritation of which has caused inflammatory thickening in the surrounding parts. How many patients have been allowed to die of such a condition in the belief that they had malignant disease of the abdomen no one can tell. But for such patients a gastro jejunostomy holds out the prospects of a speedy and perfect cure."

Mr. Power said that these were composite pictures in the sense that no individual patient necessarily presented all the characters indicated, though all resemble each other sufficiently closely to enable a diagnosis to be made with comparative ease as soon as one became alive to the occurrence of duodenal ulceration and its sequelæ. He then proceeded to examine in detail the signs, symptoms and treatment of duodenal ulcer, and arrived finally at the conclusions that: (*a*) Duodenal ulcers are not very uncommon, that they are usually single and occur more frequently in men than in women. (*b*) Duodenal ulcers may perforate and cause acute symptoms or they may heal and by cicatrisation lead to symptoms of chronic duodenal obstruction. (*c*) The sequelæ of a healed ulcer may be so remote that the symptoms are mistaken for those due to cancer of the pylorus and the patient is allowed to drift from bad to worse under the erroneous notion that he is bound to die. (*d*) There is no means of recognising the existence of a duodenal ulcer in many cases, until it perforates or until the results of its cicatrisation become manifest. (*e*) The treatment of duodenal ulceration consists (i) in the direct suture of a perforated ulcer, the prognosis being less favourable than in similar cases of perforation of the stomach; (ii) the performance of gastro-jejunostomy in cases of dilated stomach due to duodenal constriction, the prognosis being the most favourable of all the conditions for which this operation is done at the present time.

